# Antibacterial Activity of Rationally Designed Antimicrobial Peptides

**DOI:** 10.1155/2020/2131535

**Published:** 2020-04-08

**Authors:** Marius B. Tincho, Thureyah Morris, Mervin Meyer, Ashley Pretorius

**Affiliations:** ^1^Bioinformatics Research Group (BRG), DST/Mintek Nanotechnology Innovation Centre–Biolabels Node, Department of Biotechnology, Faculty of Natural Sciences, University of the Western Cape, Bellville 7535, South Africa; ^2^Food Toxicology Laboratory, Department of Medical Bioscience, Faculty of Natural Sciences, University of the Western Cape, Bellville 7535, South Africa; ^3^DST/Mintek Nanotechnology Innovation Centre–Biolabels Node, Department of Biotechnology, Faculty of Natural Sciences, University of the Western Cape, Bellville 7535, South Africa

## Abstract

Many infectious diseases are still prevalent in the world's populations since no effective treatments are available to eradicate them. The reasons may either be the antibiotic resistance towards the available therapeutic molecules or the slow rate of producing adequate therapeutic regimens to tackle the rapid growth of new infectious diseases, as well as the toxicity of current treatment regimens. Due to these reasons, there is a need to seek and develop novel therapeutic regimens to reduce the rapid scale of bacterial infections. Antimicrobial Peptides (AMPs) are components of the first line of defense for prokaryotes and eukaryotes and have a wide range of activities against Gram-negative and Gram-positive bacteria, fungi, cancer cells, and protozoa, as well as viruses. In this study, peptides which were initially identified for their HIV inhibitory activity were further screened for antibacterial activity through determination of their kinetics as well as their cytotoxicity. From the results obtained, the MICs of two AMPs (Molecule 3 and Molecule 7) were 12.5 *μ*g/ml for *K. pneumoniae* (ATCC 700603) and 6.25 *μ*g/ml for *P*. *aeruginosa* (ATCC 22108). The two AMPs killed these bacteria rapidly *in vitro*, preventing bacterial growth within few hours of treatment. Furthermore, the cytotoxic activity of these two peptides was significantly low, even at an AMP concentration of 100 *μ*g/ml. These results revealed that Molecule 3 and 7 have great potential as antibacterial drugs or could serve as lead compounds in the design of therapeutic regimens for the treatment of antibiotic-resistant bacteria.

## 1. Introduction

The human body is equipped with a defence mechanism, which enables it to eradicate foreign bodies and/or pathogenic organisms [1, 2]. However, the inability of the human defence system to defend itself following a microbial invasion of the immune system will ultimately result in complete immunity breakdown, hence giving way for the entrance of other pathogenic organisms into the body.

While most bacteria cohabitate with human cells without causing any harm and disruption, some common infectious pathogens that would cause diseases may include the Methicillin-resistant *Staphylococcus aureus* (MRSA) strains, *Candida albicans*, Herpes simplex, *Mycobacterium avium complex* (MAC), *Mycobacterium tuberculosis, Klebsiella pneumoniae*, and *Pseudomonas aeruginosa*, just to name a few [3]. *S. aureus*, *K. pneumoniae, E. coli*, and *P. aeruginosa* are examples of bacteria that have serious clinical and medical implications in individuals, and these bacteria account for the major causes of nosocomial infections worldwide [4, 5]. In addition, these pathogenic microbes have been cited as the major causative agents for many infections such as skin infections, respiratory infections, and other major illnesses. In some instances, these infections can lead to life-threatening diseases such as pneumonia, meningitis, toxic shock syndrome, and bacteremia [5–7].

Besides the fact that the immune system of some individuals cannot withstand any bacterial infection, the main problem is that the currently available antibiotics used to eradicate these pathogenic microbes are ineffective. Such ineptitude of new antibiotics is a result of the microbial resistance towards them. Furthermore, immunocompetent individuals infected with *S. aureus*, *P. aeruginosa*, and/or *K*. *pneumoniae* have also demonstrated low susceptibility to these drugs due to antibiotic resistance genes [8–11].

The lack of effective antibacterial antibiotics to inhibit the infectious pathogens and to stop the ability of these microorganisms to replicate has encouraged microbiologists and clinical pathologists to embark on a journey in search of alternative remedies to treat such microbial infections. Some antimicrobial peptides have proven to be good sources of antibacterial activity [12–17].

A number of these peptides have been commercially developed and are available on the market. Some examples include the US FDA-approved Polymixin B-Collistin-Colomycin (prodrug) and Daptomycin (Cubicin) which are used to treat skin infections [18, 19]. Likewise, the implementation of AMPs has yielded substantial results in demonstrating their activity against Gram-positive and Gram-negative bacteria, protozoa, fungi, viruses, and specifically HIV [20–26].

In a previous study, we were able to identify and validate AMPs with potent anti-HIV activity [27]. Some of these AMPs were also used as ligands for the diagnosis of HIV [28, 29]. A follow-up study describing the *in-silico* site-directed mutagenesis of the parental anti-HIV AMPs to increase their binding affinity was carried out, and the anti-HIV activity demonstrated that these mutated AMPs have increased anti-HIV activity as compared to their parental AMPs. Furthermore, the broad neutralizing ability and the mechanism of action of these anti-HIV AMPs were demonstrated as well (unpublished data, a manuscript is in preparation). However, in the current study, we screened these peptides against a number of Gram-positive and Gram-negative bacteria to investigate if these antimicrobial peptides could have antibacterial activity besides their anti-HIV activity. The antibacterial experiment was also to confirm the efficacy of the bioinformatics method used to identify peptides with diverse microbial activities. The results showed that some of the AMPs have moderate activity against Gram-positive bacteria. However, the peptides exhibit potent activity against the Gram-negative bacteria such as *Klebsiella pneumoniae* (ATCC 700603) and *Pseudomonas aeruginosa* (ATCC 22108) that are clinical antibiotic-resistant strains. Moreover, some peptides with antibacterial activity proved to have a fast killing kinetic within a reasonable time against these bacteria. Their cytotoxicity activity toward human cell lines was also significantly low.

## 2. Materials and Methods

### 2.1. Bacterial Strains

The antibacterial activity of the putative antimicrobial peptides was carried out on *Staphylococcus aureus* spp., a Gram-positive bacterium obtained from the American Type Culture Collection (ATCC), with the *S. aureus* spp. comprising of the methicillin-sensitive *S. aureus* (ATCC 25923) and the methicillin-resistant *S. aureus* (ATCC 33591) strains. The other bacterial strains tested were *K. pneumoniae* (ATCC 700603) and *P. aeruginosa* (ATCC 22108) which are Gram-negative bacteria.

### 2.2. Antimicrobial Peptide Compounds

Five putative anti-HIV AMPs were utilized for the antibacterial assay (Table 1), and their physicochemical properties were characterized (Table 2). The AMPs were identified as described in a previous work by Tincho [27]. The selected AMPs were obtained from GL Biochem Ltd. (Shanghai 200241, China), and they were chemically synthesized using the solid-phase method and they were purified to > 98 % by reverse-phase High-Pressure Liquid Chromatography.

### 2.3. Preparation of Antimicrobial Peptides and Positive Control Concentration

A stock solution of AMPs was prepared by dissolving an amount of AMPs in sterile distilled water (dH_2_O), and the various AMP working concentrations used in the microtiter broth dilution assay were prepared in two-fold serial dilutions starting at a concentration of 500 *μ*g/ml to 1.5625 *μ*g/ml. Ampicillin was utilized as the positive control in this assay and a working stock solution of 100 *μ*g/ml was prepared in distilled water.

### 2.4. Antimicrobial In Vitro Assays

#### 2.4.1. Antibacterial Activities and Minimum Inhibitory Concentration (MIC)

The microtiter broth dilution method was employed to measure the antibacterial activity of the AMP's. The assay was performed according to the standards and guidelines as stipulated in the Clinical and Laboratory Standards Institute (CLSI) [30]. In brief, the test microorganisms (MRSA (ATCC 33591), MSSA (ATCC 25923), *K. pneumoniae* (ATCC 700603), and *P*. *aeruginosa* (ATCC 22108)) were grown to the midlogarithmic phase in Tryptic Soy Broth (TSB) on a shaker set at 37°C and shaking at 150 rpm. The turbidity was adjusted to a 0.5 McFarland standard with a final volume of 10 ml. From the stock of prepared AMP solutions, various working peptide concentrations (500 *μ*g/ml to 1.5625 *μ*g/ml) were prepared, and a mixture of the bacteria with the peptides was incubated in a 96 well flat bottom plate for 24 h at 37°C. Following 24 h incubation, 40 *μ*l of 2-(4-iodophenyl)-3-(4-nitrophenyl)-5-phenyl-2*H*-tetrazolium (INT) was added to each well and incubated again for 3 h. Absorbance readings were taken at 620 nm on a microtiter plate reader (Omega®POLARstar BMG Labtech, USA).

All the antibacterial assays of the AMPs were prepared in triplicate, and the experiments were repeated three times to ensure the reproducibility of the testing. Absorbance results were exported into an Excel file, where they were transformed into a percentage, in a Normalizing process. The MIC of each peptide was defined as the concentration resulting in about 90% killing of the initial inoculums.

#### 2.4.2. Growth Inhibitory Assay

The growth curves for *K. pneumoniae* (ATCC 700603) and *P*. *aeruginosa* (ATCC 22108) treated with Molecule 3 and Molecule 7 over time were determined based on measurements taken at 620 nm at each collection time. Different concentrations of the peptide were added to the tested strains (*K. pneumonia* and *P. aeruginosa*), which were cultured in 96 well plates using the same method as used for the determination of the antimicrobial activity of the AMPs and the MIC.

### 2.5. Cytotoxicity

The cytotoxicity activity of the AMPs was measured by using the 3-(4,5-dimethylthiazol-2-yl)-2,5- diphenyltetrazolium bromide (MTT) assay as described by Freimoser [31]. In brief, after the trypsinisation of HEK293T and HepG2 cells, 1 × 10^4^ cells/well were seeded in a 96-well sterile plate and grown to confluence in Dulbecco's modified Eagle's medium containing 10% fetal calf serum. Molecule 3 and Molecule 7 were each added to the wells at different concentrations (10 *μ*g/ml, 25 *μ*g/ml, 50 *μ*g/ml, and 100 *μ*g/ml). Cells treated with 6% DMSO served as positive control. After 48 h after treatment with the peptides, 20 *μ*l of MTT solution was added to each well, and the plates were incubated in a humidified incubator for 3 h in 5% CO_2_ at 37°C. After incubation, the media was removed and dimethyl sulfoxide (DMSO) (100 *μ*l per well) was added into each well and the plate was again incubated in a shaker at 37°C, 5% CO_2_ incubator for 10 min. The plates were gently swirled for 10 min at room temperature to dissolve the precipitate. The absorbance was determined using a multiplate reader (Omega®POLARstar BMG Labtech, USA) at a wavelength of 570 nm, 600 nm, and 630 nm.

The assay was repeated three times to confirm the reproducibility of the experiment. The final absorbance of the treated cells was obtained by subtracting the background absorbance of the multi-well plate at 630 nm and from the 570 nm measurements. The percentage cell viability was calculated using the following formulae:

(1) $$\begin{array}{c} \mathrm{Cell}\;\mathrm{viability}\;\left(\%\right)=\frac{\left({\text{OD}}_{570}-{\text{OD}}_{630}\right)\left(\text{Treated sample}\right)}{\left({\text{OD}}_{570}-{\text{OD}}_{630}\right)\left(\text{Untreated control}\right)}\times 100. \end{array}$$

Absorbance results were exported into an Excel file, where they were transformed into a percentage, in a process called normalizing.

### 2.6. Statistical Analysis

The data presented are means ± SD obtained from at least three independent experiments. The statistical analysis and the cytotoxicity values of each putative AMP were performed using GraphPad Prism software (GraphPad software, San Diego, CA, USA). The differences between the means were considered to be significant if *p* < 0.05 according to Prism's two-way ANOVA test. Standard error bars represent the s.d. of the mean (±s.d.) and ^*∗*^*p* < 0.05 denoted the significant differences between the means of the untreated and treated cells.

## 3. Results

### 3.1. The In Vitro Antibacterial Activity of the Antimicrobial Peptides

The antibacterial activities of the identified AMPs were studied using the microtiter method as prescribed by the CLSI standard. Initial screening of these peptides showed that two AMPs (Molecule 3 and Molecule 7) have more effective antibacterial activity on Gram-negative bacteria, as compared to Gram-positive bacteria (Table 3). Further experiments to determine the MICs of the tested AMPs with antibacterial activity showed that the MICs of Molecule 3 were 12.5 *μ*g/ml for *K. pneumoniae* (ATCC 700603) and 6.25 *μ*g/ml for *P*. *aeruginosa* (ATCC 22108). The same MICs values were observed for Molecule 7 with *K. pneumoniae* (ATCC 700603) and *P*. *aeruginosa* (ATCC 22108).

The data suggest that the Gram-negative bacteria were more sensitive to Molecule 3 and Molecule 7, with more effective inhibitory activities toward the antibiotic-resistant pathogens than the traditional antibiotic drugs. However, Gram-positive bacteria (MRSA (ATCC 33591) and MSSA (ATCC 25923)) were both insensitive to these peptides (Molecule 3 and 7).

### 3.2. Growth Inhibitory Activities of Molecule 3 and Molecule 7 on the Bacteria

**Figure 1:**
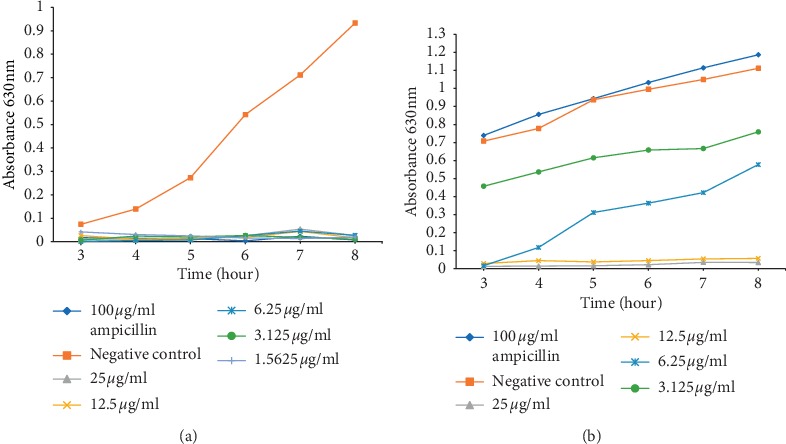
Growth-inhibitory effects of Molecule 3 against *P. aeruginosa* (ATCC 22108) and *K. pneumoniae* (ATCC 700603). (a) The time curve of Molecule 3 against *P. aeruginosa* (ATCC 22108). (b) The time curve of Molecule 3 against *K. pneumoniae* (ATCC 700603). The tested strain was cultured in 96-well plates, and the OD_620_ was measured at each time point. The error bars indicate standard deviations from the mean.

**Figure 2:**
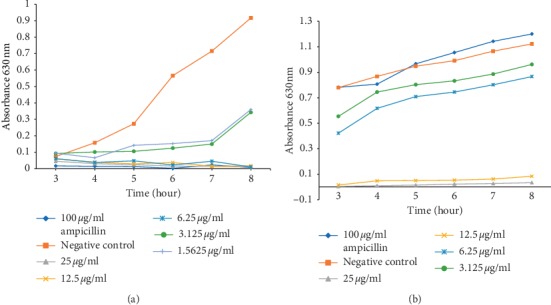
Growth-inhibitory effects of Molecule 7 against *P. aeruginosa* (ATCC 22108) and *K. pneumoniae* (ATCC 700603). (a) The time curve of Molecule 7 against *P. aeruginosa* (ATCC 22108). (b) The time curve of Molecule 7 against *K. pneumoniae* (ATCC 700603). The tested strain was cultured in 96-well plates, and the OD_620_ was measured at each time point. The error bars indicate standard deviations from the mean.

In addition to the determined MICs, we investigated the rate at which the peptides influence the bacterial growth over time (Figures 1 and 2). The OD_620_ was measured from 3 to 8 h after treatment with a microplate reader, with Ampicillin as a positive control. It was found that both peptides (Molecule 3 and molecule 7) could inhibit *K. pneumoniae* (ATCC 700603) and *P. aeruginosa* (ATCC 22108) growth at concentrations of 25 *μ*g/ml and lower, at a very short period of time after treatment (Figures 1 and 2). It was noticed that even with an AMP concentration lower than the determined MIC, Molecule 3 and 7 could still completely inhibit *P. aeruginosa* (ATCC 22108) growth 3–8 h after treatment (Figures 1(a) and 2(a)). However, Molecule 3 was able to inhibit *P. aeruginosa* at all the concentrations and for the duration of the experiment, while Molecule 7 could not inhibit *P. aeruginosa* growth at concentrations lower than the MIC (6.25 *μ*g/ml) (Figure 2(a)). Similarly, Molecule 7 could not suppress the growth of *K. pneumoniae* (ATCC 700603) at concentrations lower than the respective MIC values obtained for Molecule 7 on these organisms (Figure 2(b)). Molecule 3 was able to suppress the growth of *K. pneumoniae* (ATCC 700603) at concentrations equal to the MIC (12.5 *μ*g/ml) and 2x MIC higher than the MIC. At concentration of 6.25 *μ*g/ml, Molecule 3 was able to suppress growth for a period of 4 h (Figure 1(b)). Furthermore, the positive control, ampicillin, could inhibit the growth of *P. aeruginosa* (ATCC 22108) at a concentration of 100 *μ*g/ml. However, the same concentration of ampicillin (100 *μ*g/ml) failed to inhibit the growth of *K. pneumoniae* (ATCC 700603). The possibility to inhibit the growth of *K. pneumoniae* (ATCC 700603) and *P. aeruginosa* (ATCC 22108) at different AMPs concentrations demonstrated that the inhibitory effect of these AMPs is dose-dependent.

### 3.3. Cytotoxicity Activity of Molecule 3 and Molecule 7 on HEK293T and HepG2 Cell Lines

**Figure 3:**
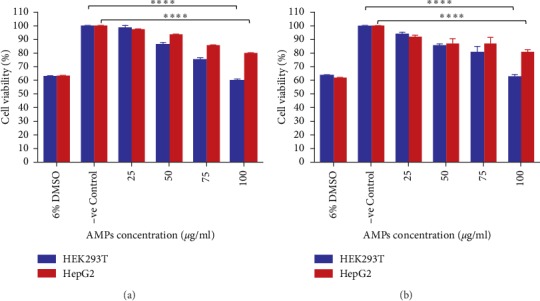
Cytotoxicity of Molecule 3 and Molecule 7 to mammalian cells. (a) Cytotoxicity of Molecule 3 to HepG2 and HEK293T cell lines. (b) Cytotoxicity of Molecule 7 to HepG2 and HEK293T cell lines. Cytotoxicity was measured with an MTT assay. The concentrations of Molecule 3 and Molecule 7 ranged from 0 to 100 *μ*g/ml. The positive control was 6.0% DMSO. The error bars indicate standard deviations from the mean. *∗∗∗∗* Statistical significance (*p* < 0.001) compared to negative control.

The cytotoxicity of Molecule 3 and Molecule 7 against mammalian cells HEK293T and HepG2 were tested by an MTT method (Figures 3(a) and 3(b)), using a dose-response assay (concentrations of 25, 50, 75, and 100 *μ*g/ml were used). Molecule 3 and Molecule 7 induced a dose-dependent decrease in the viability of the two-cell lines tested in this study. Only a moderate decrease in cell viability was observed for both peptides (Molecules 3 and molecule 7), even at the highest peptide concentration of 100 *μ*g/ml, with a cell viability of 79.5% for the HepG2 cell line. However, considerable (*p* < 0.0001) cytotoxicity as compared to the untreated cell line (negative control) was observed when HEK 293T cell line was treated with Molecule 3 and Molecule 7 with a cell viability of only 59.29%. Nevertheless, when the peptide concentration used is 25 *μ*g/ml, both cell lines inhibition are more than 90% (Figures 3(a) and 3(b)). But, at this concentration, Molecule 3 and 7 effectively inhibited the growth of the Gram-negative bacteria, including *K. pneumonia* (ATCC 700603) and *P. aeruginosa* (ATCC 22108), showing that these peptides would be less toxic to normal human cells.

## 4. Discussion

The medical and pharmaceutical industries are in a race to discover and develop novel drugs that would serve as potent antimicrobials to combat several diseases. This journey is a time-consuming process, demands a lot of funds, and sometimes ends up being rejected at the clinical trial stages due to numerous side effects. Another major problem with new antibiotics developed in the past decade is the increase in bacterial resistance to available antibiotics, which is caused by abuse of antibiotic. Potent antimicrobial peptide-based drugs can possibly counter the antibiotic-resistance.

In the quest to search for novel peptide-based antibiotics, we screened a number of AMPs for antibacterial activity using the microtiter method. From the results, it was shown that only two antimicrobial peptides were able to completely inhibit the growth of *P. aeruginosa* and *K. pneumonia* at the respective AMP concentration of 6.5 *μ*g/ml and 12.5 *μ*g/ml (Table 3). Even the highest AMP concentration of 500.0 *μ*g/ml was not able to inhibit up to 50% of both treated *Staphylococcus* spp. even for the methicillin-sensitive *Staphylococcus aureus* (MSSA). This was also true for the methicillin-resistant *Staphylococcus aureus* (MRSA). Only moderate bacterial inhibitions were observed 24 h after treatment of both Staphylococcus *spp*. with 500.0 *μ*g/ml AMP concentrations (Data not shown).

From the results, we observed that Molecule 3 and 7 have similar Lys and Arg percentage composition and these two amino acids are the most common residues in these two peptides among the tested antimicrobial peptides (Table 1 and Table 2); and these amino acids have been documented to be important for the cationic function of AMPs [32–34]. Furthermore, the two AMPs have similar isoelectric potential and both AMPs have the best positively charged parameters as compared to the other tested peptides (Table 2). Additionally, the presence of the positively charged residues and a hydrophobicity of 43% are excellent elements that enable these peptides to have better antibacterial activity as opposed to the three other peptides [35–37].

The differences observed in antibacterial activity against Gram-negative and Gram-positive bacteria could be explained by the differences in cell wall chemistry. Gram-negative bacteria have an additional outer membrane, which makes them more resistant to conventional antibiotics [38, 39]. Thus, the greater activity of the AMPs against Gram-negative bacteria is a promising result. The use of novel molecules such as AMPs on antibiotic-resistant bacteria could, therefore, serve as an additional template for drugs design against these antibiotic-resistant bacteria. The results obtained in this study could also be explained by the presence of a single layer of heavy peptidoglycan volume in the membrane of Gram-positive bacteria, which could prevent the destruction of the *Staphylococcus* spp. by the AMPs. In contrast, the Gram-negative bacteria have a light peptidoglycan layer with an extra outer membrane; thus, the tested bacteria (*P. aeruginosa* (ATCC 22108) and *K. pneumoniae* (ATCC 700603)) were sensitive to the AMPs. Hence, it can be said that the role of sugar moieties (N-linked sugars, peptidoglycans, and lipopolysaccharides) on the microorganism's membrane is to protect them against neutralizing antibodies and antibiotics in certain situations [40–42].

Although the result points out that 100 *μ*g/ml ampicillin could not inhibit the growth of *K. pneumonia*, moreover, it was observed that this bacterium seemed to grow more than the untreated bacterium when treated with 100 *μ*g/ml ampicillin. Lin et al. reported the same observation in 2013, when *K. pneumonia* was treated with 5 mg/ml of *Fructus mume* seems to grow more than the untreated *K. pneumonia* after the second hour after experiment; later, the treated bacterium stop growing than the untreated one and surpass the untreated bacterium from the 24 h after treatment [43]. The patterns of the untreated *K. pneumonia* and the treated *K. pneumonia* with 5 mg/ml of *Fructus mume* reported in this study are similar to the curve recorded for the untreated *K. pneumonia* and the *K. pneumonia* treated with 100 *μ*g/ml Ampicillin (Figures 1(b) and 2(b)).

The ability of these two antimicrobial peptides to inhibit the Gram-negative bacteria *P. aeruginosa* (ATCC 22108) and *K. pneumoniae* (ATCC 700603) also demonstrates that the model developed for the antimicrobial peptides is to be taking into consideration as future tool for the design and the identification of potent peptides with broad microbial activities. Although the initial bioinformatics models were developed to identify AMPs with HIV activity, it does limit these AMPs just to the HIV activity since it has previously been showed that a single antimicrobial peptide may exert multiple antimicrobial activities [44–50].

Many AMPs have been proven to have antibacterial activity on selected pathogenic microbes [12–17, 49], and even though most of these AMPs have not passed the final stage of clinical trials and only few are used [50–52], the current antibacterial activity exhibited by the two AMPs look promising since the peptides are more active than a known antibiotic, ampicillin on *K. pneumoniae* (ATCC 700603) (Figures 1(b) and 2(b)). Furthermore, Molecule 3 and Molecule 7 kill *P. aeruginosa* (ATCC 22108) and *K. pneumoniae* (ATCC 700603) at a fast rate and still maintain their activity for a long period after treatment. This ability, thus, qualifies Molecule 3 and molecule 7 as being bactericidal. In addition, both AMPs (Molecule 3 and Molecule 7) have a reduced toxicity to the HEK293T cell line with a lesser cytotoxicity of the AMPs observed on HepG2 cell line (a type of human liver cancer cell line). The selective toxicity of the AMPs is probably due to differences in the membranes between the mammalian cells and the bacteria.

Despite the fact that the mechanism of action by which Molecule 3 and Molecule 7 exert their antibacterial activity has not yet been established, the bactericidal ability of these AMPs could be a result of either using the Barrel–stave mechanism, the carpet mechanism [53], or the toroidal pore mechanism [24]. The barrel–stave mechanism is more likely since most of our peptides are *α*-helical, *β*-sheet peptides, extended with *α*-helical structure, and extended with *β*-sheet structure; and it has been found that most *α*-helical or *β*-sheet AMPs use this mechanism to exert their activity on pathogens [54]. The antibacterial activity displayed by both Molecule 3 and Molecule 7 against *P. aeruginosa* (ATCC 22108) and *K. pneumoniae* (ATCC 700603) and their low toxicity on HEK293T and HepG2 cell lines suggest that these two AMPs may be good lead compounds for the design of effective antibacterial drugs of peptide origin.

## 5. Conclusion

The exploration of additional and new medications to combat many infectious diseases is the ultimate goal of most research facilities and/or pharmaceutical companies around the world. It has become imperative that we find novel lead molecules that can be utilized for the development of these new drugs to which the pathogens are sensitive. Here, a set of peptides, previously shown to have HIV activity, have demonstrated rapid bactericidal activity against pathogenic Gram-negative bacteria. Additionally, both AMPs with antibacterial activity are less toxic on HEK293T and HepG2 cell lines, even at concentration 4X higher to the bacterial MIC. These results demonstrate that these peptides are clinically important and are an excellent template to design an anti-infective drug. Further works will be to test the peptide activities on clinical strains, establish the mechanism of action of the antimicrobial peptides, and perform in vivo testing of the peptides to confirm their antibiotic potential.

## Figures and Tables

**Table 1:** Sequences of the tested antimicrobial peptides.

	Peptides sequences
Molecule 1	CLRYKKPECQSDWQCPGKKRCCPDTCGIKCLDPVDTPNPTRRKPGKCPVTYGQCLMLNPPNFCEMDGQCKRDLKCCMGM
Molecule 3	RWKLFKKIEKVGRNVRDGLIKAGPAIAVIGQAKSLGK
Molecule 7	RWKIFKKIEKMGRNIRDGIVKAGPAIEVLGSAKAIGK
Molecule 8	CLKSGAICHPVFCPRRYKQIGTCGLPGTKCCKKP
Molecule 10	WNPFKELEKAGQRVRDAIISAKPAVDVVGQATAIIK

**Table 2:** Physicochemical characterization of the putative antimicrobial peptides.

	Mass (Da)	Lys %	Arg %	Cys %	Isoelectric point	Net charge	Total hydrophobic ratio (%)	Protein-binding potential (Boman index)
Molecule 1	8903.716	11.39	6.33	16	8.37	+6	34	2.17 kcal/mol
Molecule 3	4040.889	18.92	8.11	0.00	11.86	+8	43	1.37 kcal/mol
Molecule 7	4073.940	18.92	8.11	0.00	11.46	+7	43	1.45 kcal/mol
Molecule 8	3670.552	14.71	5.88	17.65	9.60	+8	38	1.07 kcal/mol
Molecule 10	3908.564	11.11	5.56	0.00	10.33	+2	47	1.33 kcal/mol

**Table 3:** The MICs of the AMPs using the microtiter during the screening process.

Peptides	MIC (*μ*g/ml)			
	MRSA	MSSA	*K. pneumoniae*	*P. aeruginosa*
Molecule 1	>500	>500	500	>500
Molecule 3	>500	>500	**12.5**	**6.25**
Molecule 7	>500	>500	**12.5**	**6.25**
Molecule 8	>500	>500	>500	>500
Molecule 10	>500	>500	500	>500
Ampicillin	>100	>100	>100	100

## Data Availability

The data used to support the findings of this study are available from the corresponding author upon request.
